# Association between achievement motivation and anxiety symptoms among college students

**DOI:** 10.3389/fpsyg.2026.1868871

**Published:** 2026-06-11

**Authors:** Jiayu Liu, Chunting Wang, Haoge Bai, Hongwei Zhang, Jiale Li, Yixuan Li, Xin Dong, Xuanhe Zhang, Fangfang Xu, Hao Sun

**Affiliations:** 1 Clinical Medical College, Jining Medical University, Jining, Shandong, China; 2 Fengtai District Center for Disease Control and Prevention, Beijing, China; 3 School of Educational Science, Jiangsu Normal University, Xuzhou, Jiangsu, China; 4 College of Medical Imaging and Laboratory, Jining Medical University, Jining, Shandong, China; 5 College of Mental Health, Jining Medical University, Jining, Shandong, China; 6 Clinical Medical College (Affiliated Hospital), Jining Medical University, Jining, Shandong, China; 7 School of Mental Health (Research Institute of Mental Health), Jining Medical University, Jining, Shandong, China

**Keywords:** achievement motivation, anxiety symptoms, college students, educational psychology, emotional disorders

## Abstract

**Background:**

Emotional problems are prevalent among college students, and many experience low levels of achievement motivation. This study investigated the correlation between achievement motivation and anxiety symptoms among medical students from Jining Medical University in Jining, China.

**Method:**

In September 2025, a cross-sectional study was performed among 3,173 college students in Jining City. Data were collected through electronic questionnaires consisting of three core components: sociodemographic characteristics, the Achievement Motivation Scale, and the 7-item Generalized anxiety symptoms Scale (GAD-7). Statistical analyses were conducted using SPSS 27.0 software, including univariate analysis, multiple logistic regression analysis and correlation analysis, to identify factors associated with anxiety symptoms and achievement motivation, as well as to verify the correlation between these two variables.

**Results:**

A total of 3,173 questionnaires were distributed, and 2,827 valid questionnaires were collected, with an effective response rate of 89.09%; among the 2,827 college students surveyed, 28.76% reported mild anxiety symptoms, 4.89% reported moderate symptoms, and 1.31% reported severe symptoms. Achievement motivation analysis revealed that 59.43% of the participants exhibited high levels, while 35.58% showed low levels. Multivariate logistic regression analysis revealed that the severity of anxiety was significantly associated with being left behind in childhood, family income, parental health status, chronic diseases (respiratory/digestive systems), satisfaction with major, and academic planning (*p* < 0.05), but not with age, height, weight, gender, or BMI. The severe anxiety group exhibited more pronounced health risk behaviors and academic adaptation issues, necessitating targeted interventions. Related research has shown that anxiety severity was significantly positively correlated with failure avoidance motivation, and negatively correlated with success pursuit motivation.

**Conclusion:**

Our study reveals that anxiety symptoms are significantly associated with individual patterns of achievement motivation. Achievement motivation is closely correlated with anxiety symptoms among college students: fear of failure is linked to higher anxiety levels, while pursuit of success is related to lower anxiety levels. These findings suggest that motivational orientation adjustments may be an important target in mental health interventions, though causal direction remains to be established. To promote college students’ mental health, schools should carry out adaptive motivation training programs to help students shift from a “failure-avoidance” motivational orientation toward a “success-pursuit” orientation. Meanwhile, targeted academic planning guidance should be integrated into mental health intervention, as academic planning showed a protective role against severe anxiety in the current study. While simultaneously strengthening psychological support and academic planning guidance for students with backgrounds such as left-behind experiences or family financial difficulties.

## 1 Introduction

Recently, mental health issues among college students have become increasingly prominent (Beiter et al., 2015). Mental disorders in young population have become a significant global public health concern (Erskine et al., 2016). According to the Mental Health Blue Book released by the Chinese Academy of Sciences, titled the “Report on National Mental Health Development in China (2021–2022),” the prevalence of psychological disorders among college students is 22.8% (Wu et al., 2020). The detection rates of depression and anxiety risk were approximately 21.48 and 45.28%, respectively (Ahmed et al., 2022). Moreover, another research effort has found that the mental health status of undergraduate students in key universities is less favorable, a situation that requires attention (Sheldon et al., 2021). Recognized extensively as a mental disorder, anxiety symptoms have also been proven to be risk factors for several diseases, including hypertension, diabetes, peptic ulcer disease, and sleep disorders (Niles et al., 2015). During the COVID-19 pandemic, the prevalence of anxiety symptoms among college students rosen sharply, with perceived stress levels reaching 43.7% (Yuan et al., 2023). This increase has emerged as a crucial determinant of academic achievement and the overall quality of life. Achievement motivation, a core psychological trait that motivates individuals to pursue goals, is significantly linked to academic performance and mental health (Ma et al., 2024).

Achievement motivation is defined as the motivation to participate in activities that are both meaningful and challenging (Sano and Kyougoku, 2016). Besides Atkinson’s expectancy-value theory, contemporary theories including Achievement Goal Theory, Self-Determination Theory, and Control-Value Theory also provide important theoretical foundations for understanding the interplay between motivational orientation and anxiety symptoms in student populations (Atkinson and Feather, 1966). Moreover, students with insufficient achievement motivation may develop anxiety due to academic procrastination, which is often caused by a lack of clear goal orientation. Students with low levels of achievement motivation who often lack a clear goal orientation may be more likely to experience anxiety that is associated with academic procrastination (Seema, 2024). This seemingly contradictory phenomenon suggests that the association between achievement motivation and anxiety is nuanced and may depend on contextual or individual factors, warranting further investigation into its boundary conditions and underlying mechanisms.

College student anxiety arises from a combination of factors including academic pressure, social challenges, and self-expectations (Jones et al., 2018). Although some studies have uncovered partial associations between achievement motivation and anxiety symptoms, the complex relationship between them remains unclear. For example, inadequate sample sizes and improper choice of self-report questionnaires can result in inaccurate findings (Kukshinov et al., 2024). Additionally, there is still a need for further research on gender differences and mediating mechanisms, such as time management and academic pressure. Therefore, exploring the association between the two not only aids in understanding the dynamic mechanisms of mental health, but also provides a scientific basis for mental health interventions in higher education institutions.

## 2 Method

### 2.1 Participants and procedures

This study adopted a convenience sampling method to recruit undergraduate students from multiple majors and grades of Jining Medical University. Anonymous online questionnaires were uniformly distributed during April to October 2025. Invalid questionnaires with regular responses and missing key items were excluded to ensure data quality. A total of 3,173 university students were invited to participate and 2,827 valid questionnaires were ultimately analyzed, resulting in a response rate of 89.10%. Each question was completed independently by the students. This study was approved by the Ethics Committee of Jining Medical University. Written informed consent was obtained from all participants.

### 2.2 Assessments

Researchers used a self-designed scale to collect data on four aspects of college students: core demographic profile, family background information, health status, and academic factors. Detailed information is presented in Supplementary Table 1. Height and weight were measured on-site by designated personnel using standard recommended methods, and BMI was calculated and classified according to the standards of the “Guidelines for the Prevention and Control of Overweight and Obesity in Chinese Adults” as follows: BMI = weight (kg)/height (m)^2^. BMI < 18.50 kg/m^2^ is classified as underweight, 18.50 ≤ BMI < 24.00 kg/m^2^ as normal weight, 24.00 ≤ BMI < 28.00 kg/m^2^ as overweight, and BMI ≥ 28.00 kg/m^2^ as obesity.

The Achievement Motivation Scale (AMS) is a widely utilized instrument designed for application in educational and vocational research. Originally developed in 1970 by Norwegian psychologists T. Gjesme and R. Nygard at the University of Oslo, the scale was translated into Chinese in 1989 (Ye and Hagtvet, 1989). Based on the expectancy-value theory of achievement motivation, which posits distinct outcomes for positive (success-oriented) and negative (failure-avoidant) motivational tendencies, the AMS comprises two subscales: “motive for success” (Ms) and “motive to avoid failure” (Mf) (Man et al., 1994). It is applicable in educational and vocational research contexts. The AMS consists of 30 items, with 15 items for each dimension. Participants rated their agreement with each item using a 4-point Likert scale: 1 = strongly disagree, 2 = disagree, 3 = agree, and 4 = strongly agree. Higher scores indicate stronger endorsement of the content, with elevated scores on the Ms. and Mf subscales reflecting heightened corresponding tendencies. The total AMS score is usually calculated as the difference between the total Ms. score and the total Mf score, ranging from −45 to 45, with higher values indicating stronger achievement motivation. Higher values denote stronger overall achievement motivation: Ms. > Mf: Dominant success-oriented motivation. Ms. = Mf: Moderate achievement motivation. Ms. < Mf: Predominant failure-avoidant motivation. In the current study, the Cronbach’s *α* reliability for the total AMS scale was 0.78, for the Ms. subscale was 0.89, and for the Mf subscale was 0.92, which indicates that the scales have good internal consistency (Supplementary Table 2). In addition to categorical grouping, the total score of achievement motivation was further analyzed as a continuous variable to avoid information loss and improve statistical sensitivity.

The Seven-item Generalized anxiety symptoms Scale (GAD-7) was used to assess anxiety symptoms. The GAD-7 is a self-report questionnaire designed to screen for anxiety symptoms in the general population (Spitzer et al., 2006). This questionnaire contains seven items, each rated on a 4-point scale from 0 (not at all) to 3 (nearly every day), with total scores ranging from 0 to 21. Scores of 0–4 indicate no anxiety symptoms, 5–9 indicating mild anxiety, 10–14 indicating moderate anxiety, and 15–21 indicating severe anxiety. In this study, the reliability of the scale, as measured by Cronbach’s *α*, was 0.90 in this study.

### 2.3 Statistical analysis

The collected data were analyzed using IBM SPSS Statistical software for Windows version 27.0 (George and Mallery, 2021), and the normality of all quantitative variables was assessed using the Kolmogorov–Smirnov test (Steinskog et al., 2007). Continuous variables, such as age, height, and weight, are expressed as median (P25, P75), and categorical variables, such as sex and BMI, are expressed as frequencies and percentages (*n* [%]). We first used univariate analysis of variance, Kruskal-Wallis H tests (for non-normally distributed continuous variables), and chi-square tests (for categorical variables) to systematically analyze differences in anxiety levels across different variables. Subsequently, Covariates included in the multivariate logistic regression model were selected primarily based on theoretical and empirical evidence, supplemented by variables with *p* < 0.1 in the univariate analysis to avoid data-driven bias and model instability to control for potential confounding factors and to systematically analyze the combined effects of multiple variables on anxiety levels, ensuring the robustness of the conclusions (Ataman et al., 1996; Bursac et al., 2008). Results were considered statistically significant when *p* < 0.05. Prior to multivariate logistic regression, anxiety symptoms were categorized into three levels: no anxiety (GAD-7 score 0–4), mild anxiety (5–9), and moderate-to-severe anxiety (10–21). The moderate and severe groups were combined because (a) clinically, both levels warrant professional psychological intervention and exhibit continuity in symptom severity, and (b) statistically, the small sample size of the severe anxiety subgroup (*n* = 37) would compromise model stability and statistical power if analyzed separately. The association between anxiety levels and achievement motivation was analyzed using cross-tabulation chi-square tests, and measures of association strength, such as Phi, Cramer’s V, and Kendall’s tau-b, were calculated. The significance level for tests was set at α = 0.05.

## 3 Results

### 3.1 Demographic characteristics of participants

Descriptive characteristics of the participants are presented in Table 1. This study ultimately included 2,827 college students, consisting of 1,309 males (46.30%) and 1,518 females (53.70%), with a median age of 19.00 (18.00, 20.00) years. Among the participants, 827 were only children (29.25%), and 2,000 had at least one sibling (70.75%). A total of 1,839 participants (65.05%) reported no anxiety symptoms, 813 participants (28.8%) reported mild anxiety symptoms, 138 participants (4.88%) reported moderate anxiety symptoms, and 37 participants (1.3%) reported severe anxiety symptoms. A total of 1,680 participants (59.4%) reported high levels of achievement motivation (Ms > Mf), 141 (5.0%) reported moderate levels of achievement motivation (Ms = Mf), and 1,006 (35.6%) reported low levels of achievement motivation (Ms < Mf).

**Table 1:** Demographic characteristics of participants.

Variables	*N* (%)
Sex	
Male	1,309 (46.30)
Female	1,518 (53.70)
BMI	
Normal	1,686 (59.64)
Underweight	482 (17.05)
Overweight	475 (16.80)
Obesity	184 (6.51)
Family structure	
Nuclear family	2,165 (76.58)
Others	662 (23.42)
Only child	
Yes	827 (29.25)
No	2,000 (70.75)
Paternal education level	
Primary school or below	263 (9.30)
Secondary school or equivalent	1,524 (53.91)
College/tertiary education or above	1,040 (36.79)
Maternal education level	
Primary school or below	417 (14.75)
Secondary school or equivalent	1,469 (51.96)
College/tertiary education or above	941 (33.29)
Annual family income (RMB^a^)	
Below 80,000	1,389 (49.13)
80,000-150,000	978 (34.59)
Above 150,000	460 (16.27)
Monthly living expenses (RMB^a^)	
Below 1,000	228 (8.07)
1,000-2,000	2,357 (83.37)
Above 2,000	242 (8.56)
Left-behind experience	
Yes	460 (16.27)
No	2,367 (83.73)
Health status	
Self	
No	454 (16.06)
Yes	2,373 (83.94)
Mother	
No	904 (31.98)
Yes	1,923 (68.02)
Father	
No	677 (23.95)
Yes	2,150 (76.05)
Hypertension	
Self	
No	2,797 (98.94)
Yes	30 (1.06)
Mother	
No	2,374 (83.98)
Yes	453 (16.02)
Father	
No	2,613 (92.43)
Yes	214 (7.57)
Diabetes mellitus	
Self	
No	2,822 (99.82)
Yes	5 (0.18)
Mother	
No	2,698 (95.44)
Yes	129 (4.56)
Father	
No	2,762 (97.70)
Yes	65 (2.30)
Coronary artery heart disease	
Self	
No	2,827 (100.00)
Yes	0 (0.00)
Father	
No	2,794 (98.83)
Yes	33 (1.17)
Mother	
No	2,789 (98.66)
Yes	38 (1.34)
Cancer	
Self	
No	2,827 (100.00)
Yes	0 (0.00)
Father	
No	2,810 (99.40)
Yes	17 (0.60)
Mother	
No	2,805 (99.22)
Yes	22 (0.78)
Respiratory disease	
Self	
No	2,730 (96.57)
Yes	97 (3.43)
Father	
No	2,765 (97.81)
Yes	62 (2.19)
Mother	
No	2,788 (98.62)
Yes	39 (1.38)
Digestive system disease	
Self	
No	2,634 (93.17)
Yes	193 (6.83)
Father	
No	2,701 (95.54)
Yes	126 (4.46)
Mother	
No	2,737 (96.82)
Yes	90 (3.18)
Anxiety symptoms	
Self	
No	2,785 (98.51)
Yes	42 (1.49)
Father	
No	2,814 (99.54)
Yes	13 (0.46)
Mother	
No	2,804 (99.19)
Yes	23 (0.81)
Major	
Medicine	2,715 (96.04)
Others	112 (3.96)
Academic satisfaction with chosen discipline	
No	181 (6.40)
Yes	2,646 (93.60)
On-time graduation status	
No	1,329 (47.01)
Yes	1,498 (52.99)
Student leadership experience	
No	1,909 (67.53)
Yes	918 (32.47)
Scholarship attainment status	
No	970 (34.31)
Yes	1,857 (65.69)
Extracurricular community engagement	
No	1,958 (69.26)
Yes	869 (30.74)
Professional competency competitions	
No	1,963 (69.44)
Yes	864 (30.56)
Postgraduate education aspirations	
No	391 (13.83)
Yes	2,436 (86.17)

BMI, body mass index. ^a^1 US $ ≈ 7.2 RMB.

### 3.2 Univariate analysis results

Supplementary Table 3 delineates the variable coding scheme and operational definitions employed in the statistical analysis.

A total of 2,827 participants were included in this study and were categorized into four groups based on their GAD-7 scores: no anxiety symptoms group (*n* = 1,839, 65.05%), mild anxiety group (*n* = 813, 28.76%), moderate anxiety group (*n* = 138, 4.88%), and severe anxiety group (*n* = 37, 1.31%). Differences in demographic characteristics, health status, and academic-related variables among the four groups were analyzed using the Kruskal-Wallis H test (for continuous variables) or chi-square test (for categorical variables), with the results presented in Table 2. No statistically significant differences were found among the four groups for any of the continuous variables (age, height, weight) (*p* > 0.05).

**Table 2:** Univariate analysis in anxiety symptoms.

Variables	Anxiety symptoms				H/χ^2^ value	*p* Value
	No anxiety	Mild	Moderate	Severe		
N (%)	1893 (65.05)	813 (28.76)	138 (4.88)	37 (1.31)		
Continuous variables	*M* (P25, P75)				H value^b^	
Age (years)	19.00 (18.00, 20.00)	19.00 (18.00, 20.00)	19.00 (18.00, 20.00)	19.00 (18.00, 20.00)	2.036	0.565
Height (m)	1.70 (1.65, 1.78)	1.70 (1.64, 1.77)	1.70 (1.65, 1.75)	1.68 (1.63,1.74)	3.256	0.354
Weight (kg)	62.00 (54.00, 73.00)	62.00 (54.00, 70.00)	60.00 (53.00, 72.00)	58.00 (50.00, 69.00)	3.742	0.291
Categorical variables	*N* (%)				χ^2^ value^c^	
Sex						
Male	868 (47.20)	366 (45.02)	62 (44.93)	13 (35.14)	3.095	0.377
Female	971 (52.80)	447 (54.98)	76 (55.07)	24 (64.86)		
BMI						
Normal	1,080 (58.73)	509 (62.61)	76 (55.07)	21 (56.76)	12.070	0.209
Underweight	309 (16.80)	138 (16.97)	26 (18.84)	9 (24.32)		
Overweight	324 (17.62)	119 (14.64)	29 (21.01)	3 (8.11)		
Obesity	126 (6.85)	47 (5.78)	7 (5.07)	4 (10.81)		
Family structure						
Nuclear family	1,425 (77.49)	618 (76.01)	99 (71.74)	23 (62.16)	7.082	0.069
Others	414 (22.51)	195 (23.99)	39 (28.26)	14 (37.84)		
Only child						
Yes	551 (29.96)	222 (27.31)	44 (31.88)	10 (27.03)	2.485	0.478
No	1,288 (70.04)	591 (72.69)	94 (68.12)	27 (72.97)		
Paternal education level						
Primary school or below	161 (8.75)	81 (9.96)	16 (11.59)	5 (13.51)	3.510	0.743
Secondary school or equivalent	1,003 (54.54)	434 (53.38)	70 (50.72)	17 (45.95)		
College/tertiary education or above	675 (36.70)	298 (36.65)	52 (37.68)	15 (40.54)		
Maternal education level						
Primary school or below	239 (13.00)	149 (18.33)	23 (16.67)	6 (16.22)	13.838	**0.032** ^**d**^
Secondary school or equivalent	980 (53.29)	403 (49.57)	69 (50.00)	17 (45.95)		
College/tertiary education or above	620 (33.71)	261 (32.10)	46 (33.33)	14 (37.84)		
Annual family income (RMB^a^)						
Below 80,000	867 (47.15)	425 (52.28)	78 (56.52)	19 (51.35)	13.242	**0.039**
80,000 - 150,000	650 (35.35)	279 (34.32)	38 (27.54)	11 (29.73)		
Above 150,000	322 (17.51)	109 (13.41)	22 (15.94)	7 (18.92)		
Monthly living expenses (RMB^a^)						
Below 1,000	129 (7.01)	77 (9.47)	17 (12.32)	5 (13.51)	10.744	0.097
1,000 - 2,000	1,549 (84.23)	672 (82.66)	108 (78.26)	28 (75.68)		
Above 2,000	161 (8.75)	64 (7.87)	13 (9.42)	4 (10.81)		
Left-behind experience						
Yes	264 (14.36)	152 (18.70)	35 (25.36)	9 (24.32)	18.595	**<0.001**
No	1,575 (85.64)	661 (81.30)	103 (74.64)	28 (75.68)		
Good health						
Self						
No	239 (13.00)	140 (17.22)	58 (42.03)	17 (45.95)	107.170	**<0.001**
Yes	1,600 (87.00)	673 (82.78)	80 (57.97)	20 (54.05)		
Father						
No	545 (29.64)	284 (34.93)	52 (37.68)	23 (62.16)	25.462	**<0.001**
Yes	1,294 (70.36)	529 (65.07)	86 (62.32)	14 (37.84)		
Mother						
No	388 (21.10)	218 (26.81)	58 (42.03)	13 (35.14)	39.180	**<0.001**
Yes	1,451 (78.90)	595 (73.19)	80 (57.97)	24 (64.86)		
Hypertension						
Self						
No	1,823 (99.13)	802 (98.65)	135 (97.83)	37 (100.0)	3.324	0.344
Yes	16 (0.87)	11 (1.35)	3 (2.17)	0 (0.00)		
Father						
No	1,557 (84.67)	674 (82.90)	118 (85.51)	25 (67.57)	8.989	**0.029**
Yes	282 (15.33)	139 (17.10)	20 (14.49)	12 (32.43)		
Mother						
No	1,719 (93.47)	743 (91.39)	117 (84.78)	34 (91.89)	15.676	**<0.001**
Yes	120 (6.53)	70 (8.61)	21 (15.22)	3 (8.11)		
Diabetes mellitus						
Self						
No	1,836 (99.84)	811 (99.75)	138 (100.00)	37 (100.00)	0.550	0.908
Yes	3 (0.16)	2 (0.25)	0 (0.00)	0 (0.00)		
Father						
No	1,756 (95.49)	780 (95.94)	128 (92.75)	34 (91.89)	3.834	0.280
Yes	83 (4.51)	33 (4.06)	10 (7.25)	3 (8.11)		
Mother						
No	1,801 (97.93)	790 (97.17)	135 (97.83)	36 (97.30)	1.496	0.683
Yes	38 (2.07)	23 (2.83)	3 (2.17)	1 (2.70)		
Coronary artery heart disease						
Self						
No	1,839 (100.00)	813 (100.00)	138 (100.00)	37 (100.00)	N/A	N/A
Yes	0 (0.00)	0 (0.00)	0 (0.00)	0 (0.00)		
Father						
No	1,820 (98.97)	802 (98.65)	137 (99.28)	35 (94.59)	6.525	0.089
Yes	19 (1.03)	11 (1.35)	1 (0.72)	2 (5.41)		
Mother						
No	1,818 (98.86)	805 (99.02)	129 (93.48)	37 (100.00)	29.763	**<0.001**
Yes	21 (1.14)	8 (0.98)	9 (6.52)	0 (0.00)		
Cancer						
Self						
No	1,839 (100.00)	813 (100.00)	138 (100.00)	37 (100.00)	N/A	N/A
Yes	0 (0.00)	0 (0.00)	0 (0.00)	0 (0.00)		
Father						
No	1,827 (99.35)	809 (99.51)	137 (99.28)	37 (100.00)	0.502	0.918
Yes	12 (0.65)	4 (0.49)	1 (0.72)	0 (0.00)		
Mother						
No	1,823 (99.13)	811 (99.75)	136 (98.55)	35 (94.59)	14.248	**0.003**
Yes	16 (0.87)	2 (0.25)	2 (1.45)	2 (5.41)		
Respiratory disease						
Self						
No	1,789 (97.28)	781 (96.06)	128 (92.75)	32 (86.49)	20.855	**<0.001**
Yes	50 (2.72)	32 (3.94)	10 (7.25)	5 (13.51)		
Father						
No	1,810 (98.42)	786 (96.68)	133 (96.38)	36 (97.30)	9.437	**0.024**
Yes	29 (1.58)	27 (3.32)	5 (3.62)	1 (2.70)		
Mother						
No	1,821 (99.02)	800 (98.04)	132 (95.65)	35 (94.59)	15.803	**0.001**
Yes	18 (0.98)	13 (1.60)	6 (4.35)	2 (5.41)		
Digestive system disease						
Self						
No	1,745 (94.89)	753 (92.62)	109 (78.99)	27 (72.97)	76.303	**<0.001**
Yes	94 (5.11)	60 (7.38)	29 (21.01)	10 (27.03)		
Father						
No	1,771 (96.30)	771 (94.83)	128 (92.75)	31 (83.78)	17.986	**<0.001**
Yes	68 (3.70)	42 (5.17)	10 (7.25)	6 (16.22)		
Mother						
No	1,787 (97.17)	787 (96.80)	128 (92.75)	35 (94.59)	8.739	**0.033**
Yes	52 (2.83)	26 (3.20)	10 (7.25)	2 (5.41)		
Anxiety symptoms						
Self						
No	1,827 (99.35)	802 (98.65)	126 (91.30)	30 (81.08)	134.665	**<0.001**
Yes	12 (0.65)	11 (1.35)	12 (8.70)	7 (18.92)		
Father						
No	1,837 (99.89)	807 (99.26)	134 (97.10)	36 (97.30)	28.323	**<0.001**
Yes	2 (0.11)	6 (0.74)	4 (2.90)	1 (2.70)		
Mother						
No	1,823 (99.13)	810 (99.63)	135 (97.83)	36 (97.30)	6.865	0.470
Yes	16 (0.87)	3 (0.37)	3 (2.17)	1 (2.70)		
Major						
Medicine	1,764 (95.92)	781 (96.06)	134 (97.10)	36 (97.30)	0.631	0.889
Others	75 (4.08)	32 (3.94)	4 (2.90)	1 (2.70)		
Academic satisfaction with chosen discipline						
Yes	1,746 (94.94)	746 (91.76)	122 (88.41)	32 (86.49)	19.470	**<0.001**
No	93 (5.06)	67 (8.24)	16 (11.59)	5 (13.51)		
On-time graduation status						
No	872 (47.42)	379 (46.62)	61 (44.20)	17 (45.95)	0.626	0.890
Yes	967 (52.58)	434 (53.38)	77 (55.80)	20 (54.05)		
Student leadership experience						
No	1,210 (65.80)	566 (69.62)	105 (76.09)	28 (75.68)	9.865	**0.020**
Yes	629 (34.20)	247 (30.38)	33 (23.91)	9 (24.32)		
Scholarship attainment status						
No	611 (33.22)	288 (35.42)	58 (42.03)	13 (35.14)	5.068	0.167
Yes	1,228 (66.78)	525 (64.58)	80 (57.97)	24 (64.86)		
Extracurricular community engagement						
No	1,244 (67.65)	592 (72.82)	97 (70.29)	25 (67.57)	7.201	0.066
Yes	595 (32.35)	221 (27.18)	41 (29.71)	12 (32.43)		
Professional competency competitions						
No	1,252 (68.08)	588 (72.32)	97 (70.29)	26 (70.27)	4.849	0.183
Yes	587 (31.92)	225 (27.68)	41 (29.71)	11 (29.73)		
Postgraduate education aspirations						
No	234 (12.72)	124 (15.25)	24 (17.39)	9 (24.32)	8.154	**0.043**
Yes	1,605 (87.28)	689 (84.75)	114 (82.61)	28 (75.68)		

BMI, body mass index; N/A, No variation in data. ^a^1 US $ ≈ 7.2 RMB. ^b^Kruskal-Wallis H test; ^c^Chi-Square test. ^d^Bold is significant.

Consistent with the present finding (*p* = 0.377), the gender distribution of anxiety symptoms did not reach statistical significance in this medical student sample, which was partly different from previous reports of higher anxiety prevalence in female students (Yeretzian et al., 2023). The homogeneous academic pressure and learning environment among medical students may narrow gender differences in anxiety. Future research could further examine the moderating effect of gender on the association between achievement motivation and anxiety. The proportion of nuclear families decreased with increasing anxiety levels (77.49% in the no anxiety group vs. 62.16% in the severe anxiety group, χ^2^ = 7.082, *p =* 0.069), although this difference was not statistically significant. The proportion of individuals with a history of being left behind was significantly higher in the moderate (25.36%) and severe anxiety groups (24.32%) compared to the no anxiety group (14.36%, χ^2^ = 18.595, *p* < 0.001). The proportion of participants whose mothers had an elementary school education or lower was higher in the mild anxiety group (18.33% vs. 13.00% in the no anxiety group, χ^2^ = 13.838, *p =* 0.032). The proportion of individuals who self-rated their health as poor significantly increased with increasing anxiety levels (13.00% in the no anxiety group vs. 45.95% in the severe anxiety group, χ^2^ = 107.170, *p* < 0.001). The proportion of participants with poor paternal health was significantly higher in the severe anxiety group (62.16% vs. 29.64% in the no anxiety group, χ^2^ = 25.462, *p* < 0.001). The proportion of individuals who self-reported respiratory diseases was highest in the severe anxiety group (13.51% vs. 2.72% in the no anxiety group, χ^2^ = 20.855, *p* < 0.001). The proportion of participants who self-reported gastrointestinal diseases significantly increased with increasing anxiety levels (5.11% in the no anxiety group vs. 27.03% in the severe anxiety group, χ^2^ = 76.303, *p* < 0.001). The proportion of individuals who Self-reported elevated anxiety symptoms was highest in the severe anxiety group (18.92% vs. 0.65% in the no anxiety group, χ^2^ = 134.665, *p* < 0.001). The proportion of participants dissatisfied with their major increased with increasing anxiety levels (13.51% in the severe anxiety group vs. 5.06% in the no anxiety group, χ^2^ = 19.470, *p* < 0.001). The proportion of individuals without leadership experience was significantly higher in the moderate (76.09%) and severe anxiety groups (75.68%) compared to the no anxiety group (65.80%, χ^2^ = 9.865, *p =* 0.020). The proportion of participants without plans for further education was highest in the severe anxiety group (24.32% vs. 12.72% in the no anxiety group, χ^2^ = 8.154, *p =* 0.043).

Variables such as BMI (χ^2^ = 12.070, *p =* 0.209), only child status (χ^2^ = 2.485, *p =* 0.478), father’s education level (χ^2^ = 3.510, *p =* 0.743), and hypertension (χ^2^ = 3.324, *p =* 0.344) did not show statistically significant differences across the groups.

### 3.3 Multivariate logistic regression analysis

Anxiety symptoms were thus categorized into three groups: no anxiety (*n* = 1,839), mild anxiety (*n* = 813), and moderate-to-severe anxiety (*n* = 175). The rationale for this combination was twofold. First, clinically, moderate and severe anxiety share similar professional psychological intervention needs and symptom severity continuity. Second, statistically, the severe anxiety subgroup contained only 37 participants; separate modeling would lead to unstable logistic regression results and insufficient statistical power. Anxiety symptoms were set as the dependent variable, and variables with *p* < 0.1 in the univariate analysis were included as covariates in the multivariate logistic regression analysis. No multicollinearity was detected among the covariates, with all variance inflation factor (VIF) values being less than 3.

This study analyzed the results of influencing factors across different levels of anxiety (mild vs. no anxiety; moderate-to-severe vs. no anxiety). The results are shown in Table 3. Family structure was found to have a protective effect on moderate-to-severe anxiety (OR = 0.68, 95% CI = 0.47–0.99, *p* = 0.042), but it was not significantly associated with mild anxiety. The experience of being left-behind did not reach statistical significance in either group (*p* > 0.05), although the odds ratio showed an increasing trend with the severity of anxiety (OR = 1.44, *p* = 0.083 for the moderate-to-severe group). A family income of less than 80,000 yuan was associated with an increased risk of mild anxiety (OR = 1.33, 95% CI = 1.00–1.75, *p* = 0.048). Good self-rated health status significantly reduced the risk of moderate-to-severe anxiety (OR = 0.47, 95% CI = 0.27–0.81, *p* = 0.006), but had no effect on mild anxiety. Gastrointestinal diseases significantly increased the risk of moderate-to-severe anxiety (OR = 2.08, 95% CI = 1.14–3.77, *p* = 0.017). A history of maternal cancer was associated with a reduced risk of mild anxiety (OR = 0.17, 95% CI = 0.04–0.78, *p* = 0.022). Self-reported anxiety symptoms was the strongest risk factor for moderate-to-severe anxiety (OR = 7.06, 95% CI = 3.07–16.23, *p* < 0.001). Higher academic satisfaction had a protective effect on both mild (OR = 0.66, *p* = 0.015) and moderate-to-severe anxiety (OR = 0.53, *p* = 0.025). The experience of being a student leader significantly reduced the risk of moderate-to-severe anxiety (OR = 0.64, 95% CI = 0.44–0.94, *p* = 0.021). Parental hypertension, respiratory diseases (except for the marginal significant association between paternal respiratory diseases and mild anxiety, OR = 1.77, *p* = 0.050), gastrointestinal diseases (excluding self-reported), and monthly living expenses were not statistically significant in either group (*p* > 0.05).

**Table 3:** Multivariable logistic regression analysis of variables (*p* < 0.1) associated with anxiety levels.

Variables	Mild anxiety *vs.* No anxiety		Moderate/Severe anxiety *vs.* No anxiety	
	OR (95%CI)	*p* value	OR (95%CI)	*p* value
Family structure	0.96 (0.79, 1.18)	0.724	0.68 (0.47, 0.99)	0.042
Left-behind experience	1.19 (0.94, 1.50)	0.149	1.44 (0.95, 2.17)	0.083
Good health				
Self	0.98 (0.69, 1.38)	0.892	0.47 (0.27, 0.81)	0.006
Mother	1.01 (0.76, 1.32)	0.970	1.08 (0.65, 1.78)	0.775
Father	0.77 (0.59, 1.01)	0.063	0.68 (0.42, 1.12)	0.133
Hypertension				
Mother	1.04 (0.76, 1.41)	0.810	0.94 (0.54, 1.63)	0.829
Father	0.96 (0.65, 1.40)	0.821	1.29 (0.70, 2.39)	0.421
Coronary artery heart disease				
Mother	0.63 (0.27, 1.47)	0.283	2.27 (0.87, 5.97)	0.096
Cancer				
Mother	0.17 (0.04, 0.78)	0.022	0.67 (0.16, 2.81)	0.580
Respiratory disease				
Self	1.28 (0.75, 2.20)	0.364	1.39 (0.67, 2.89)	0.377
Respiratory disease				
Father	1.77 (1.00, 3.15)	0.050	0.93 (0.33, 2.63)	0.895
Mother	1.07 (0.50, 2.30)	0.858	2.29 (0.85, 6.21)	0.102
Digestive system disease				
Self	1.27 (0.81, 2.00)	0.290	2.08 (1.14, 3.77)	0.017
Father	1.19 (0.77, 1.84)	0.444	1.52 (0.77, 3.02)	0.231
Mother	0.75 (0.44, 1.28)	0.289	1.19 (0.54, 2.63)	0.666
Anxiety symptoms				
Self	1.47 (0.62, 3.50)	0.382	7.06 (3.07, 16.23)	<0.001
Father	5.41 (1.03, 28.46)	0.046	5.38 (0.84, 34.51)	0.076
Academic satisfaction with chosen discipline	0.66 (0.47, 0.92)	0.015	0.53 (0.31, 0.92)	0.025
Student leadership experience	0.88 (0.73, 1.06)	0.171	0.64 (0.44, 0.94)	0.021
Postgraduate education aspirations	0.91 (0.71, 1.16)	0.435	0.81 (0.53, 1.26)	0.355
Maternal education level				
Primary school or below	1.15 (0.87, 1.52)	0.316	0.62 (0.35, 1.08)	0.089
Secondary school or equivalent	0.86 (0.71, 1.05)	0.144	0.68 (0.46, 1.00)^c^	0.047
College/tertiary education or above	1.00^b^	-	1.00^b^	-
Annual family income (RMB^a^)				
Below 80,000	1.33 (1.00, 1.75)^d^	0.048	1.00 (0.59, 1.68)	0.991
80,000–150,000	1.26 (0.95, 1.65)	0.105	0.81 (0.48, 1.37)	0.433
Above 150,000	1.00^b^	-	1.00^b^	-
Monthly living expenses (RMB^a^)				
Below 1,000	1.10 (0.70, 1.72)	0.672	1.16 (0.52, 2.56)	0.720
1,000–2,000	0.96 (0.69, 1.33)	0.786	0.88 (0.49, 1.61)	0.687
Above 2,000	1.00^b^	-	1.00^b^	-

Anxiety severity categories: “No” (0–4), “Mild” (5–9), “Moderate/Severe” (10–21); OR: odds ratio; CI: confidence interval. ^a^1 US $ ≈ 7.2 RMB. ^b^Reference. ^c^The value of 1.00 is a rounded figure; the actual value is 0.995. ^d^The value of 1.00 is a rounded figure; the actual value is 1.002.

### 3.4 Correlation between anxiety symptoms and achievement motivation

Table 4 shows that there are significant differences in the distribution of achievement motivation levels across different anxiety groups (χ^2^ = 221.219, *p* < 0.001). In the no anxiety group, the proportion of participants with strong achievement motivation was the highest (69.85%), which was significantly higher than the expected value (standardized residual = 5.30). In the moderate-to-severe anxiety group, the proportion of participants with weak achievement motivation was the highest (66.29%), which was significantly higher than the expected value (standardized residual = 6.81). In the mild anxiety group, the proportion of participants weak achievement motivation (49.08%) was also significantly higher than the expected value (standardized residual = 6.45). There was a moderate negative correlation between anxiety level and achievement motivation level (Cramer’s V = 0.198, *p* < 0.001; Kendall’s tau-b = −0.263, *p* < 0.001), indicating that higher anxiety levels were associated with weaker achievement motivation. In addition to the categorical analysis, we examined the association between the continuous total achievement motivation score (range −45 to 45) and GAD-7 scores. Spearman’s rank correlation analysis revealed a significant negative correlation between total achievement motivation and anxiety symptom severity (*ρ* = −0.31, *p* < 0.001), indicating that higher overall achievement motivation was associated with lower anxiety levels. This continuous analysis corroborates the findings from the categorical approach presented in Table 4.

**Table 4:** Association between anxiety severity and achievement motivation levels: Chi-square analysis with standardized residuals and effect size (Cramer’s V).

	Ms > Mf (%)	Ms = Mf (%)	Ms < Mf (%)
Anxiety symptoms			
No Anxiety	68.95	4.35	26.70
Mild	44.16	6.77	49.08
Moderate/Severe	30.29	3.43	66.29

Ms, motive for success; Mf, motive to avoid failure. Pearson’s χ^2^ (4) = 221.219, *p* < 0.001; Cramer’s V = 0.198; Kendall’s tau-b = −0.263, *p* < 0.001. Standardized residuals: No anxiety: Ms > Mf (+5.30), Ms = Mf (−1.22), Ms < Mf (−6.39); Mild anxiety: Ms > Mf (−5.65), Ms = Mf (2.27), Ms < Mf (6.45); Moderate/Severe anxiety: Ms > Mf (−5.00), Ms = Mf (−0.92), Ms < Mf (6.81).

## 4 Discussion

### 4.1 Main findings

Medical students face multiple challenges during their academic journey, including heavy coursework and high career development pressure. Some medical students exhibit reduced learning motivation, blurred professional identity, and decreased interpersonal interaction, which may in severe cases trigger emotional disorders such as depression and anxiety. Therefore, it is of great research value to explore the potential influencing factors of anxiety symptoms, as well as the association between achievement motivation and anxiety (Awadalla et al., 2024) within the medical student population.

#### 4.1.1 Achievement motivation was significantly negatively correlated with anxiety symptoms

The present study investigated the association between achievement motivation and anxiety symptoms among Chinese college students, revealing several key findings. These findings not only contribute to the existing literature on achievement motivation and anxiety but also provide insights into the complex interplay between these constructs in a specific cultural context.

Achievement motivation, as conceptualized by Atkinson’s expectancy-value theory, comprises two dimensions: motivation to succeed (Ms) and motivation to avoid failure (Mf) (Atkinson and Feather, 1966; Man et al., 1994). The results of this study indicated that these two dimensions have significantly different associations with anxiety symptoms. Specifically, a stronger motivation to succeed is associated with lower levels of anxiety, whereas a higher motivation to avoid failure is linked to more severe anxiety symptoms. This finding aligns with Atkinson’s theory, which posits that success-oriented goal behaviors may be associated with enhanced psychological resilience, whereas fear of failure may be associated with exacerbated psychological distress (Mubarak et al., 2022). The total achievement motivation score was significantly negatively correlated with anxiety symptoms. This suggests that when individuals’ achievement motivation is predominantly driven by their motivation to succeed, their anxiety levels are likely to be lower. This finding is consistent with previous research highlighting the protective role of positive goal pursuit in mental health (Ariani, 2017). The negative correlation between total achievement motivation and anxiety underscores the importance of fostering a success-oriented mind-set in academic settings to mitigate anxiety symptoms among college students.

Emerging evidence indicates that psychological resilience, mindfulness, cognitive appraisal, and emotional regulation play important roles in modulating the association between motivational orientation and anxiety. Recent research has shown that mindfulness training can reduce psychological threat appraisals—the perception that situational demands exceed coping resources—which in turn may buffer against the anxiety effects of performance pressure. Benson-Greenwald et al. (2024) found that mindfulness training increased students’ perceived resources and reduced psychological threat in introductory physics courses, with effects persisting three months post-intervention. Similarly, Pelakh (2024) demonstrated that mindfulness-induced reductions in psychological threat mediated anxiety relief during problem-solving tasks. Additionally, the dynamic interplay among autonomous motivation, psychological resilience, and flow states has been shown to support sustained engagement and emotional regulation under evaluative pressure. A recent case study of a Chinese world champion athlete revealed that as motivation progressively internalized toward more autonomous forms, resilience-related resources facilitated flow under pressure, and recurring flow experiences in turn reinforced motivational internalization and psychological resilience. Integrating these perspectives helps interpret the complex relationship between achievement motivation and anxiety symptoms among medical students.

#### 4.1.2 The influence of demographic variables on anxiety symptoms among medical students

Childhood left-behind experience, family income, parental health status, chronic diseases (respiratory/digestive systems), major satisfaction, and academic planning had statistically significant effects on the severity of anxiety, whereas age, height, weight, gender, and BMI did not show statistical significance. The severe anxiety group exhibited more pronounced health risk behaviors and academic adaptation problems, necessitating targeted interventions.

Although sex differences in anxiety symptoms were not statistically significant in the univariate analysis, the trend of higher anxiety levels among females compared to males was consistent with other studies (Yeretzian et al., 2023). One study found that there is a significant positive correlation between achievement motivation and anxiety levels among females, while a negative correlation was observed among males. This finding suggests that gender may play a moderating role in the relationship between achievement motivation and anxiety (Balogun et al., 2017). This trend may reflect societal and cultural pressures that differentially impact males and females as well as potential differences in coping mechanisms between genders. For instance, societal expectations may place greater emphasis on academic success for females, leading to higher levels of stress and anxiety (Trevethan et al., 2021). Additionally, females may be more likely to internalize stress, resulting in increased anxiety symptoms. Further research is needed to explore the underlying mechanisms of these gender differences and develop targeted interventions to address the specific needs of female college students.

The study also identified several significant covariates related to anxiety symptoms, including family socioeconomic factors (e.g., income and parental health status) and personal development intentions (e.g., plans for further education). These findings highlight the multifactorial nature of anxiety in academic settings. Family socioeconomic status (SES) has long been recognized as a critical factor that influences mental health outcomes (Yang et al., 2022). Lower family incomes and adverse parental health conditions may create additional stressors that contribute to higher anxiety levels among college students. For example, financial strain can lead to concerns about tuition fees and future employment prospects, whereas parental health issues may cause emotional distress and caregiving responsibilities (Jessop et al., 2020).

Personal developmental intentions, such as plans for further education, also emerged as significant predictors of anxiety symptoms. The pressure to excel academically and secure a place in graduate programs can be a substantial source of stress among college students (Kantor et al., 2023). The competitive nature of academic environments and high expectations associated with academic success may contribute to heightened anxiety levels. Moreover, the uncertainty surrounding future career paths and the perceived importance of academic achievement in securing desirable employment opportunities can exacerbate anxiety symptoms (Posselt and Lipson, 2016).

This study’s findings have several important implications for both research and practice. First, they underscore the need for a comprehensive approach to address anxiety symptoms among college students. Interventions should not only focus on enhancing achievement motivation but also on the broader context of students’ lives, including family socioeconomic factors and personal development intentions (Zhang and Wang, 2022). For example, providing financial aid and support services for students from low-income families, as well as counseling and mental health resources to help students cope with stress related to academic performance and future career planning, could be beneficial (Cardozo et al., 2020).

Second, this study highlighted the importance of sex-specific interventions. Given the trend of higher anxiety levels among females, it is crucial to develop targeted programs that address the unique challenges faced by female college students (Vitagliano et al., 2023). This may include initiatives aimed at reducing societal pressure and promoting healthy coping mechanisms.

This study suggests several avenues for future research. Longitudinal studies could provide valuable insights into the temporal relationships among achievement motivation, anxiety symptoms, and other relevant factors. Further research may also introduce psychological variables such as coping strategies to explain why individuals with similar achievement motivation profiles present different anxiety outcomes.

Additionally, research exploring the underlying mechanisms of sex differences in anxiety symptoms could inform the development of more effective interventions (Regehr et al., 2013). Furthermore, studies examining the impact of specific socioeconomic factors and personal development intentions on anxiety symptoms could help refine existing interventions and develop new strategies to support college students’ mental health (Vitagliano et al., 2023).

In conclusion, this study provides a comprehensive examination of the relationship between achievement motivation and anxiety symptoms among Chinese college students. The findings revealed significant associations between achievement motivation and anxiety symptoms, while also suggesting that cultivating a success-oriented mindset is closely related to the alleviation of anxiety. This study also underscores the multifactorial nature of anxiety in academic settings, emphasizing the need for a comprehensive approach to addressing this issue. Future research should further explore the underlying mechanisms of these relationships and develop targeted interventions to support college students’ mental health.

### 4.2 Limitations

This study has several limitations. First, as with all cross-sectional studies, the simultaneous measurement of independent and dependent variables precludes causal inference. The temporal relationship between achievement motivation and anxiety cannot be determined from the present data, and longitudinal studies are needed to establish their temporal sequence. Second, the limited sample size and the participants were exclusively recruited from one medical university, which limits the generalizability of the findings to students from comprehensive universities, non-medical majors, and other cultural and regional backgrounds. The combination of the moderate and severe anxiety groups may have masked differences between subgroups. Future studies with larger samples are needed to explore specific risk factors across different levels of anxiety severity. In particular, incorporating additional psychological variables—such as coping strategies, cognitive appraisal styles, mindfulness, and emotional regulation—may help explain why individuals with similar achievement motivation profiles exhibit different anxiety outcomes. Mediation and moderation analyses examining these mechanisms represent important directions for future research. Despite these limitations, we made efforts to control for potential confounders to minimize bias. Third, recall bias may have occurred, as participants might not have accurately or completely recalled past events and experiences. Such information bias could lead to either overestimation or underestimation of the associations between exposures and outcomes. However, we attempted to minimize this bias through careful study design and statistical adjustments. Despite these limitations, this study provides valuable insights for future research.

All data were collected via self-reported questionnaires at a single time point from the same sample, which may introduce common method variance. Future studies are recommended to adopt multi-timepoint or multi-source data collection to reduce such bias.

### 4.3 Implications for future researches

Future research should be conducted in multiple dimensions to achieve in-depth exploration. First, the sample size should be expanded to include students from different disciplines, regions, and socioeconomic backgrounds to enhance the generalizability of the conclusions. Second, longitudinal tracking or intervention experiments (e.g., adjusting motivation orientation) should be conducted to reveal the causal relationship between achievement motivation and anxiety. Simultaneously, mediating mechanisms (e.g., academic stress and coping strategies) and moderating variables (e.g., gender and cultural values) should be systematically examined to clarify the pathways and boundary conditions of the association between the two. In addition, quantitative analysis can be integrated with qualitative interviews to explore the complex experiences of the motivation-anxiety relationship (e.g., sex differences) and to focus on the regulatory effects of institutional policies (e.g., grading systems and psychological resource provision) on anxiety among students with high achievement motivation. By breaking through at multiple levels, this research can provide a basis for universities to design targeted interventions (e.g., adaptive motivation training programs), thereby collaboratively improving students’ academic performance and mental health levels.

## Data Availability

The raw data supporting the conclusions of this article will be made available by the authors, without undue reservation.
