# Supplementary material for: Association between achievement motivation and anxiety symptoms among college students
Source: Front Psychol. 2026 Jun 11;17:1868871. doi: 10.3389/fpsyg.2026.1868871 (PMC13293948; doi:10.3389/fpsyg.2026.1868871)
Supplement: Supplementary file 1 [file Supplementary_file_1.docx]

**Supplementary table 1.** Variables of self-designed questionnaire.

| **Variable classification** | **Variables** |
| --- | --- |
| **Core demographic profile** |  |
|  | Age |
|  | Sex |
|  | Height |
|  | Weight |
|  | BMI |
| **Family background information** |  |
|  | Family structure |
|  | Only-child family |
|  | Paternal education level |
|  | Maternal education level |
|  | Annual family income (RMB^a^) |
|  | Monthly living expenses (RMB^a^) |
|  | Left-behind experience |
| **Health status** |  |
|  | Good health |
|  | Hypertension |
|  | Diabetes mellitus |
|  | Coronary artery heart disease |
|  | Cancer |
|  | Respiratory disease |
|  | Digestive system disease |
|  | Anxiety disorder |
| **Academic factors** |  |
|  | Major |
|  | Academic satisfaction with chosen discipline |
|  | On-time graduation status |
|  | Scholarship attainment status |
|  | Student leadership experience |
|  | Extracurricular community engagement |
|  | Professional competency competitions |
|  | Postgraduate education aspirations |

BMI: body mass index. ^a^1 US $ ≈ 7.2 RMB.

**Supplementary table 2.** Cronbach’s α value of different scales.

| **Scale** | **Cronbach’s α** | **Items** | **N** |
| --- | --- | --- | --- |
| **AMS** | 0.78 | 30 | 2,827 |
| **Ms** | 0.89 | 15 | 2,827 |
| **Mf** | 0.92 | 15 | 2,827 |
| **GAD-7** | 0.90 | 7 | 2,827 |

AMS: the Achievement Motivation Scale; Ms: motive for success; Mf: motive to avoid failure; GAD-7: the Seven-item Generalized Anxiety Disorder Scale.

**Supplementary table 3.** Variable coding scheme.

| **Variable classification** | **Designation** | **Data type** | **Coding protocol** |
| --- | --- | --- | --- |
| **Core demographic profile** |  |  |  |
|  | Sex | Binary | 0 = Male; 1 = Female |
|  | Age | Continuous | Years (last birthday) |
|  | Height | Continuous | m |
|  | Weight | Continuous | kg |
|  | BMI | Ordinal categorical variable | 1 = Underweight;  2 = Normal;  3 = Overweight;  4 = Obesity |
| **Family background information** |  |  |  |
|  | Family structure | Binary | 1 = Nuclear family;  0 = Others |
|  | Only child | Binary | 1 = Yes; 0 = No |
|  | Paternal education level | Ordinal categorical variable | 1 = Primary school or below;  2 = Secondary school or equivalent;  3 = College/tertiary education or above |
|  | Maternal education level | Ordinal categorical variable | 1 = Primary school or below;  2 = Secondary school or equivalent;  3 = College/tertiary education or above |
|  | Annual family income (RMB^a^) | Ordinal categorical variable | 1 = Below 80,000;  2 = 80,000 - 150,000;  3 = Above 150,000 |
|  | Monthly living expenses (RMB^a^) | Ordinal categorical variable | 1 = Below 1,000;  2 = 1,000 - 2,000;  3 = Above 2,000 |
|  | Left-behind experience | Binary | 1 = Yes; 0 = No |
| **Health status** |  |  |  |
|  | Good health | Binary | 1 = Yes; 0 = No |
|  | Hypertension | Binary | 1 = Yes; 0 = No |
|  | Diabetes mellitus | Binary | 1 = Yes; 0 = No |
|  | Coronary artery heart disease | Binary | 1 = Yes; 0 = No |
|  | Cancer | Binary | 1 = Yes; 0 = No |

**Supplementary table 3 (continued).** Variable coding scheme.

| **Variable classification** | **Designation** | **Data type** | **Coding protocol** |
| --- | --- | --- | --- |
| **Health status** |  |  |  |
|  | Respiratory disease | Binary | 1 = Yes; 0 = No |
|  | Digestive system disease | Binary | 1 = Yes; 0 = No |
|  | Anxiety disorder | Binary | 1 = Yes; 0 = No |
| **Academic Factors** |  |  |  |
|  | Major | Binary | 1 = Medicine;  0 = Others |
|  | Academic satisfaction with chosen discipline | Binary | 1 = Yes; 0 = No |
|  | On-time graduation status | Binary | 1 = Yes; 0 = No |
|  | Scholarship attainment status | Binary | 1 = Yes; 0 = No |
|  | Student leadership experience | Binary | 1 = Yes; 0 = No |
|  | Extracurricular community engagement | Binary | 1 = Yes; 0 = No |
|  | Professional competency competitions | Binary | 1 = Yes; 0 = No |
|  | Postgraduate education aspirations | Binary | 1 = Yes; 0 = No |

BMI: body mass index. ^a^1 US $ ≈ 7.2 RMB.
